# Severe hematoma following the use of low molecular weight heparin in preterm neonate

**DOI:** 10.1515/crpm-2021-0086

**Published:** 2022-05-26

**Authors:** Naveed Ur Rehman Durrani, Elhindi Elfaki, Nqobile Tessa Sigola, Charlotte Tscherning, Samir Gupta, Graeme E. Glass, Phani Kiran Yajamanyum

**Affiliations:** Division of Neonatology, Department of Pediatrics, Sidra Medicine, Doha, Qatar; Department of Pediatrics, Weill Cornell Medicine – Qatar, Doha, Qatar; Durham University, Durham, UK; Department of Pediatric Surgery, Sidra Medicine, Doha, Qatar

**Keywords:** extremely low birth weight infant, hematoma, low molecular weight heparin

## Abstract

**Objectives:**

With the increased survival of preterm neonates, thromboembolic (TE) events are increasingly being recognized due to the use of indwelling catheters. It is still debatable to treat TE with low molecular weight heparin (LMWH) or follow expectant management. Despite the safety and efficacy profile about using LMWH in adults, its use in extreme preterm neonates with TE events is limited. The therapeutic level and pharmacokinetics of LMWH in the preterm population are relatively variable.

**Case presentation:**

We present a case with a severe hematoma on the left thigh following the use of LMWH, which was surgically drained and had a successful skin graft.

**Conclusions:**

This case highlights the importance of early and close monitoring of injection sites in patients treated with LMWH.

## Introduction

The survival of premature babies has improved significantly due to advances in intensive care provision, which includes administration of medications and nutrition using indwelling central venous catheters. These essential devices are associated with complications such as infections and thrombosis in major blood vessels. Although most such thrombi are non-occlusive [1], there is a potential that large thrombi can lead to life-threatening complications or obstruct the blood supply to critical organs. Thrombolysis with medications such as tissue plasminogen activator is considered very high risk, particularly in premature babies, due to the risk of significant organ bleeding. Low molecular weight heparin (LMWH), specifically enoxaparin, is used in neonates to treat thrombosis due to its ease of administration, monitoring, and favorable safety profile [2]. Common adverse effects of enoxaparin include minor bleeding at the administration site [3, 4], and rarely compartment syndrome [5], intracerebral bleeding [4], and gastrointestinal bleeding [3] may also occur. We report a premature baby who developed a large hematoma at the site of administration of enoxaparin requiring surgical evacuation and skin grafting.

## Case presentation

A male neonate was born at 24 weeks gestation, weighing 540 g. He was ventilated at birth, received two doses of surfactant. Umbilical arterial and venous catheters were placed successfully on admission. They were optimally placed as per local guidelines (UVC at T8 and UAC between T6 and T7), and UVC was replaced with a peripherally inserted central catheter on day 10.

He remained ventilated and developed spontaneous bowel perforation at 3 weeks of age. He underwent laparotomy resulting in jejunostomy formation. A workup for persistent thrombocytopenia, which he developed postoperatively, showed non-occlusive thrombi in the abdominal aorta (2.5 cm long), inferior vena cava, and hepatic vein confluence. On repeat Doppler ultrasound scan, the aortic thrombus was noted to be extending, and hence treatment with enoxaparin was recommended by a pediatric hematologist. This was initially postponed due to persistent thrombocytopenia <50 × 10^9^/L and bilateral uncomplicated intraventricular hemorrhages. Echocardiography and thrombophilia screening were unremarkable. In view of the persistent large abdominal aortic thrombus, enoxaparin was commenced at a dose of 1 mg/kg every 12 h subcutaneously via an indwelling catheter (Insuflon^®^) following a notable improvement in the platelet count (>100 × 10^9^/L).

**Figure 1:**
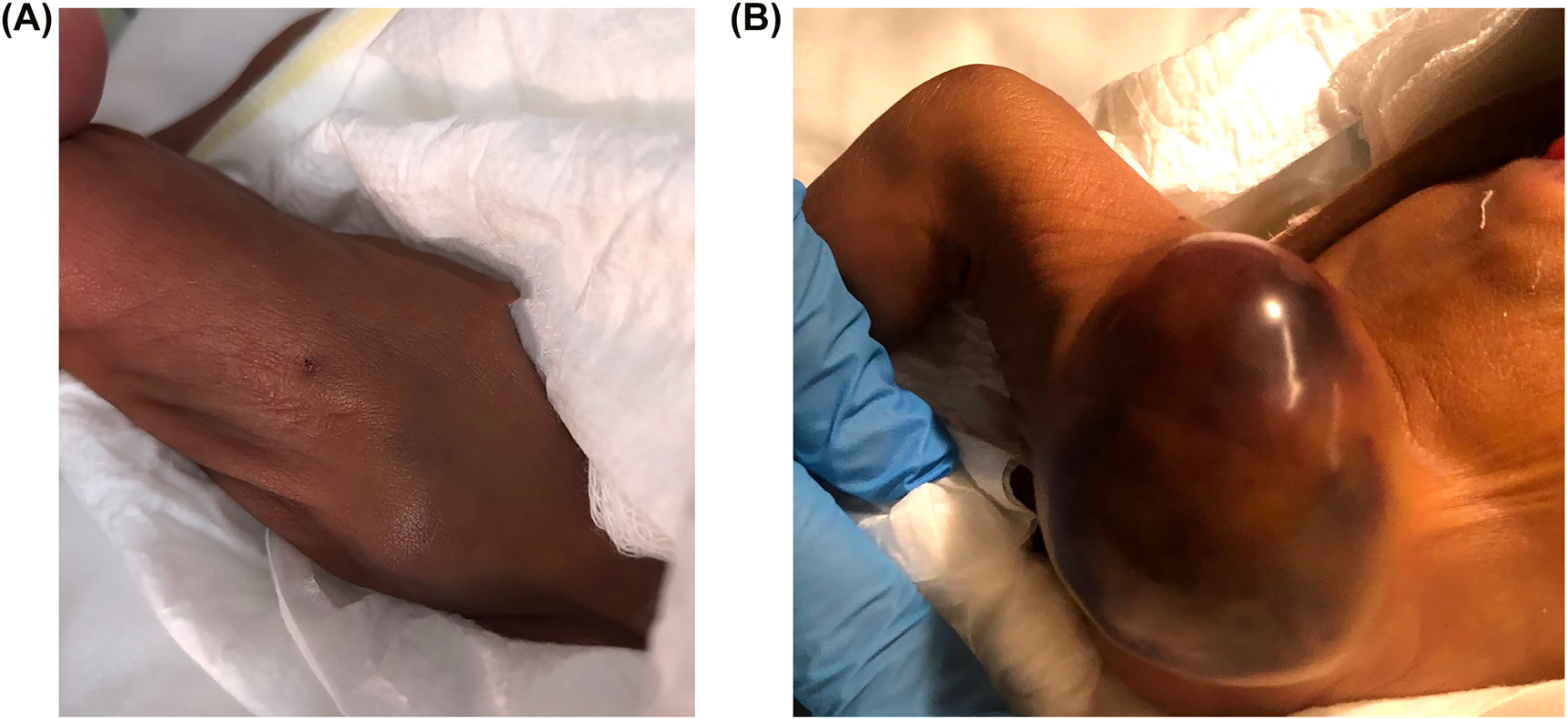
Swelling on the anterolateral aspect of the left thigh. (A) Initial minor swelling on the anterolateral aspect of the left thigh. (B) Rapid progression of initial swelling to a large hematoma.

Anti-Xa levels were measured as per standard guidelines with a target of 0.35–0.7 units/mL [4]. Still, at a maximum enoxaparin dose of 2 mg/kg/dose 12 hourly, the anti-Xa levels remained subtherapeutic. It is well recognized that premature babies require very high doses of LMWH to achieve target anti-Xa levels [3, 4]. After 21 days of enoxaparin treatment, he developed a small swelling on anterolateral aspect of the left thigh (Figure 1A), adjacent but not directly corresponding to the Insuflon site. Enoxaparin was discontinued, and bedside ultrasound revealed an extensive heterogenous subcutaneous collection measuring about 2.3 × 1.9 × 1.5 cm (Figure 1B). This swelling rapidly progressed to a large, tense hematoma over the next 12 h, necessitating emergency surgical exploration. His hemoglobin dropped from 140 to 84 g/dL, platelet count dropped to 45 × 10^9^/L, and the anti-Xa level was 0.26 U/mL at that time, with a normal coagulation screen.

**Figure 2:**
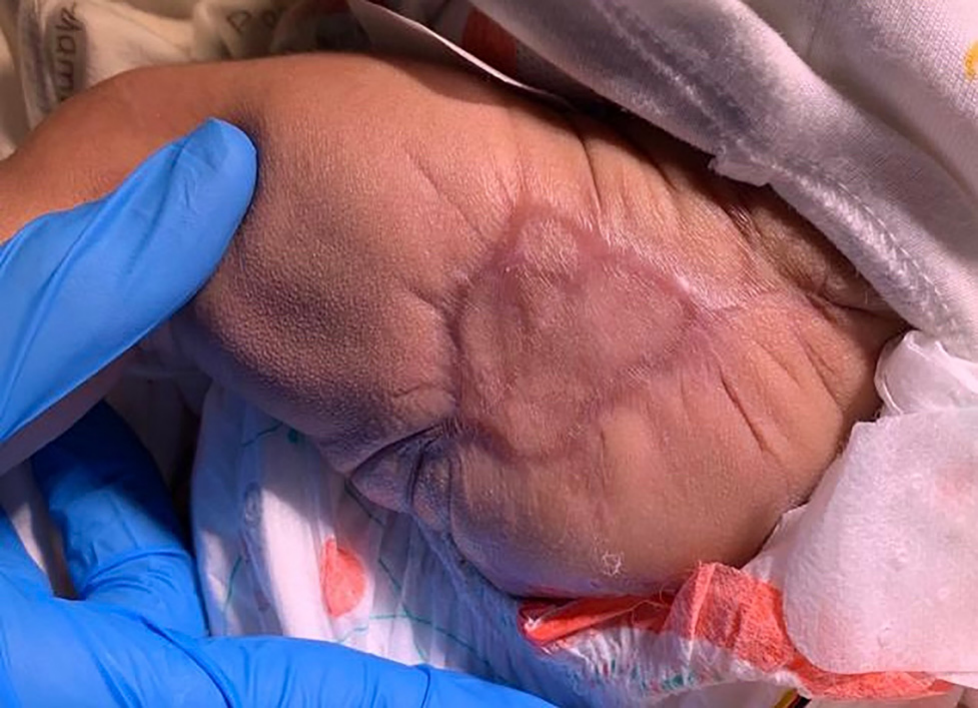
Site of large hematoma after successful skin graft.

During the surgical evacuation, the hematoma was found to be within the subcutaneous plane and superficial to the muscle fascia. While the overlying skin was speculatively redraped, it subsequently necrosed, necessitating a second surgery for debridement and closure using a full-thickness skin graft harvested as a strip from the flank [6]. This subsequently healed satisfactorily over the next 3 weeks (Figure 2). At 48 weeks of PMA, he was clinically stable and had re-anastomosis of his intestinal stomas.

## Discussion

This case demonstrates a significant complication secondary to enoxaparin therapy in a preterm infant. In our NICU (a quaternary referral center that primarily treats neonates with complex diseases), vascular thrombosis is jointly managed by NICU and pediatric hematology services. Catheter-related thrombosis is evaluated by ultrasound Doppler scanning, and enoxaparin treatment is commenced following evaluation of risks and benefits by the multi-disciplinary team.

Vascular catheter-associated thrombosis is a known complication in preterm babies [1], commonly identified as an incidental finding during ultrasound examinations. Several case series have described the incidence of catheter-related thrombosis in single centers, and there is a wide variation amongst NICUs in managing thrombosis in preterm babies – either treatment with anticoagulants, or expectant management are adopted [1, 3, 4]. To our knowledge, there have been no randomized controlled trials that have evaluated the safety and efficacy of specific therapies to manage catheter-related thrombosis in preterm neonates.

Spontaneous resolution of such thrombosis is well described [7]; however, international guidelines recommend the treatment of vascular thrombosis in preterm infants with LMWH for a total duration of 6 weeks to 3 months [8]. Van Elteren et al. reported a retrospective review which found <1% of all infants admitted to a tertiary NICU in the Netherlands had thrombotic events [7]. Of them, around 80% received LMWH, while the rest were expectantly managed. However, complete resolution of the clot was found in 86% of the untreated group and 68% of the treated group.

Van Elteren et al. [8] and Obaid et al. [5] described similar cases of a large hematoma in preterm infants following treatment with LMWH, which required decompressive surgery. In the case report described by Van Elteren et al. [8] the infant developed a significantly large hematoma when anti-Xa levels were within the therapeutic range. In contrast, the infant we describe developed hematoma even with subtherapeutic anti-Xa level. This reflects that standard anti-Xa assays may not reflect heparin concentration accurately, particularly in neonates, due to the difference in antithrombin III concentration and its rapid clearance [9]. This underestimation of heparin concentration might lead to increase side effects [10]. In a study by Malowany et al., >50% of infants receiving enoxaparin via a subcutaneous catheter experienced local side effects, but none had major bleeding [3]. In a retrospective study by Sol et al., around 5% of infants receiving LMWH had major local bleeding related to the use of subcutaneous catheters while no other major bleeding complications occurred [11].

Van Ommen et al. [1] conducted a prospective observational study of management of neonatal catheter-related thrombosis and described a pragmatic approach of close monitoring vs. active treatment. In their study, thrombi were categorized into occlusive, non-occlusive, and high risk, with management varying between thrombolysis, “wait and see”, and administration of LMWH. Major bleeding occurred in around 8% of neonates, and more than half of them suffered from significant bleeding at the site of Insuflon^®^.

## Conclusions

The use of LMWH might be justified in preterm infants to prevent extension and/or occlusive complications because of thrombi; however, the use of subcutaneous catheters for the delivery of the medication is not without its significant adverse effects, and careful consideration should be given before commencing such therapy as the spontaneous resolution of thrombi is well described. Patients receiving such treatment must be closely monitored for complications. To optimize antithrombotic management, more research is required to establish the optimal dose, safety, monitoring, and efficacy of LMWH administered via subcutaneous cannulae in preterm babies.
